# Co-infections determine patterns of mortality in a population exposed to parasite infection

**DOI:** 10.1126/sciadv.1400026

**Published:** 2015-03-20

**Authors:** Mark E. J. Woolhouse, Samuel M. Thumbi, Amy Jennings, Margo Chase-Topping, Rebecca Callaby, Henry Kiara, Marinda C. Oosthuizen, Mary N. Mbole-Kariuki, Ilana Conradie, Ian G. Handel, E. Jane Poole, Evalyne Njiiri, Nicola E. Collins, Gemma Murray, Miika Tapio, Olga Tosas Auguet, Willie Weir, W. Ivan Morrison, Loeske E. B. Kruuk, B. Mark de C. Bronsvoort, Olivier Hanotte, Koos Coetzer, Philip G. Toye

**Affiliations:** 1 Centre for Immunity, Infection and Evolution, University of Edinburgh, Ashworth Laboratories, Kings Buildings, West Mains Road, Edinburgh EH9 3JT, UK.; 2 Paul G. Allen School for Global Animal Health, Washington State University, Pullman, WA 99164–7090, USA.; 3 Royal (Dick) School of Veterinary Studies, University of Edinburgh, The Roslin Building, Easter Bush, Midlothian EH25 9RG, UK.; 4 The Roslin Institute, University of Edinburgh, The Roslin Building, Easter Bush, Midlothian EH25 9RG, UK.; 5 The James Hutton Institute, Craigiebuckler, Aberdeen AB15 8QH, UK.; 6 International Livestock Research Institute, P.O. Box 30709, Nairobi 00100, Kenya.; 7 Department of Veterinary Tropical Diseases, Faculty of Veterinary Science, University of Pretoria, Private Bag X04, Onderstepoort 0110, South Africa.; 8 School of Life Sciences, University of Nottingham, University Park, Nottingham NG7 2RD, UK.; 9 Natural Resources Institute Finland (Luke), Green technology, FI-31600 Jokioinen, Finland.; 10 Henry Wellcome Building, Institute of Biodiversity, Animal Health and Comparative Medicine, Garscube Campus, College of Medical, Veterinary and Life Sciences, University of Glasgow, Bearsden Road, Glasgow G61 1QH, UK.; 11 Division of Evolution, Ecology and Genetics, Research School of Biology, The Australian National University, Canberra, Australian Capital Territory 0200, Australia.

**Keywords:** case fatality, cattle, East Coast fever, endemic stability, Epidemiology, heterologous protection, malaria, Mathematical model, Theileria parva, vaccination

## Abstract

Many individual hosts are infected with multiple parasite species, and this may increase or decrease the pathogenicity of the infections. This phenomenon is termed heterologous reactivity and is potentially an important determinant of both patterns of morbidity and mortality and of the impact of disease control measures at the population level. Using infections with *Theileria parva* (a tick-borne protozoan, related to *Plasmodium*) in indigenous African cattle [where it causes East Coast fever (ECF)] as a model system, we obtain the first quantitative estimate of the effects of heterologous reactivity for any parasitic disease. In individual calves, concurrent co-infection with less pathogenic species of *Theileria* resulted in an 89% reduction in mortality associated with *T. parva* infection. Across our study population, this corresponds to a net reduction in mortality due to ECF of greater than 40%. Using a mathematical model, we demonstrate that this degree of heterologous protection provides a unifying explanation for apparently disparate epidemiological patterns: variable disease-induced mortality rates, age-mortality profiles, weak correlations between the incidence of infection and disease (known as endemic stability), and poor efficacy of interventions that reduce exposure to multiple parasite species. These findings can be generalized to many other infectious diseases, including human malaria, and illustrate how co-infections can play a key role in determining population-level patterns of morbidity and mortality due to parasite infections.

## Teaser

Highly protective effect of co-infections on mortality due to East Coast fever and consequences for disease epidemiology and control.

## INTRODUCTION

For many host-pathogen combinations, there is wide variability in the clinical outcome of an infection, ranging from subclinical (no symptoms or signs) through clinical illness to death. Understanding the causes and consequences of this variability is among the biggest challenges in biomedical research. Differences between individual hosts (variously attributed to genetics, environment, or demographics, especially age) and between pathogen strains may all be important (*1*, *2*). However, hosts may also differ in their history of infection by multiple other pathogen types (*3*–*5*). An influence of past or current infection with one pathogen species on the outcome of infection with another is termed heterologous reactivity.

Heterologous reactivity may have a negative impact on host health [for example, for co-infections with influenza A and *Streptococcus pneumoniae* (*6*)], but it can also have a protective effect (*7*, *8*). For viruses or bacteria, heterologous protection is often attributable to acquired immunity to one pathogen reducing susceptibility to subsequent infection with a second pathogen (*9*, *10*). However, although it has been proposed for both human schistosomes (*11*) and malaria (*12*), heterologous protection is not well established for parasites (*13*), and there have been no previous studies of its population-level impact for any parasitic disease, reflecting both a dearth of quantitative field data and the absence of an appropriate analytical framework. Here, we report how we quantified the impact of co-infections on the epidemiology and control of East Coast fever (ECF), a parasitic disease of cattle caused by the tick-borne protozoan *Theileria parva*. We then use an epidemiological model to explore the implications of heterologous protection for the epidemiology and control of ECF. The model is generic, and we expect these findings to be applicable to other parasitic diseases of cattle, humans, or other hosts.

**Fig. 1:**
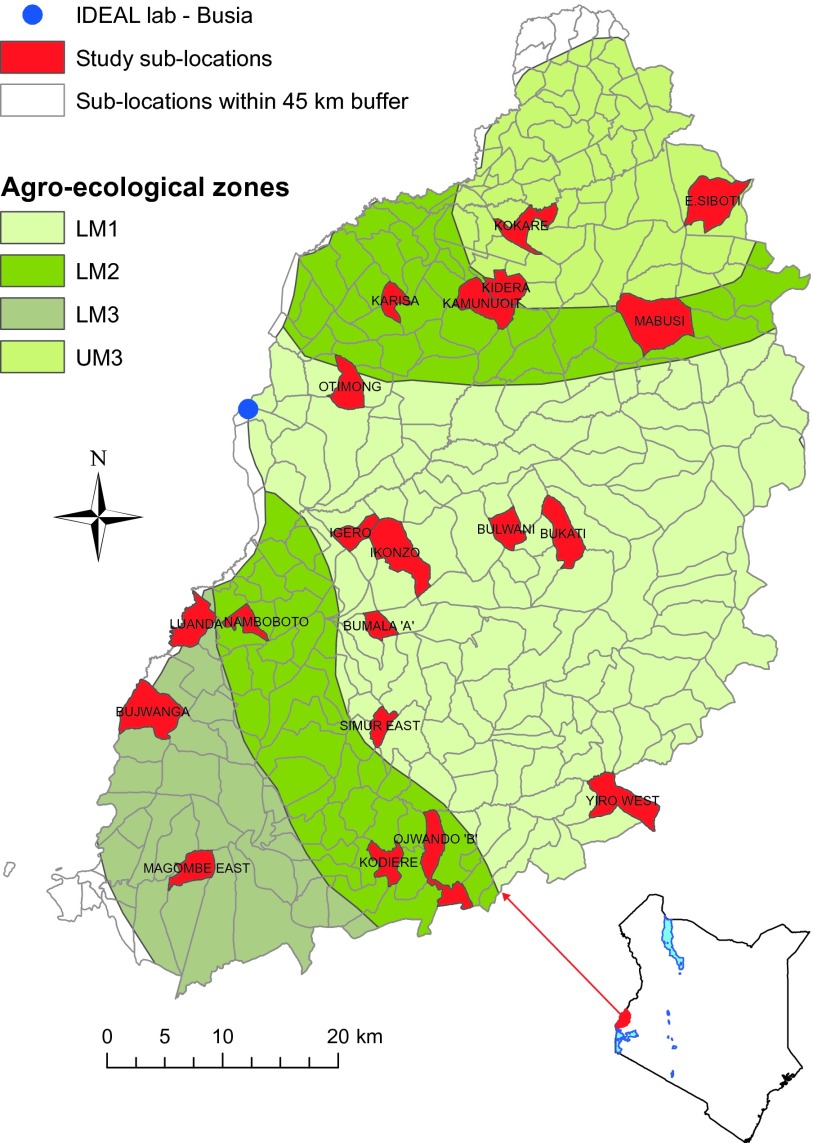
Map of study area in western Kenya. Map shows location of the study area and distinguishes different agroecological zones. Calves were recruited from 20 different sublocations (red), all falling within 45 km from the IDEAL project field laboratory in Busia town.

We studied interactions between *T. parva* and other *Theileria* species in East African shorthorn zebu calves resident in an area of western Kenya where endemic ECF imposes a substantial disease burden (*14*, *15*) (Fig. 1). This cattle breed is indigenous to East Africa and is considered more tolerant of *T. parva* infection than otherwise more productive European cattle (*16*). Cattle in this region are also routinely exposed to other, much less well-studied *Theileria* species, most commonly *Theileria mutans* and *Theileria velifera*. These two species have broadly similar life cycles to *T. parva* but differ in several key respects: they are carried by different tick vectors (*Amblyomma* spp. rather than *Rhipicephalus appendiculatus*); they proliferate primarily in erythrocytes rather than in lymphocytes; the infections are much more persistent; and, importantly, they are not associated with severe disease (*16*).

## RESULTS

### Epidemiological patterns

Our data were obtained from an observational study of a birth cohort of East African shorthorn zebu calves. Of the 454 eligible calves that survived for 1 year, serological tests indicated that 392 were exposed to *T. parva*. Of the 88 eligible calves that died, 31 were determined to have died of ECF, 1 died of turning sickness (caused by *T. parva* infection of the nervous system), and 24 others were exposed to *T. parva* before death from other or undetermined causes. Together, this gives a best estimate of 448 calves exposed to *T. parva* during their first year, of which 93% survived the infection.

**Fig. 2:**
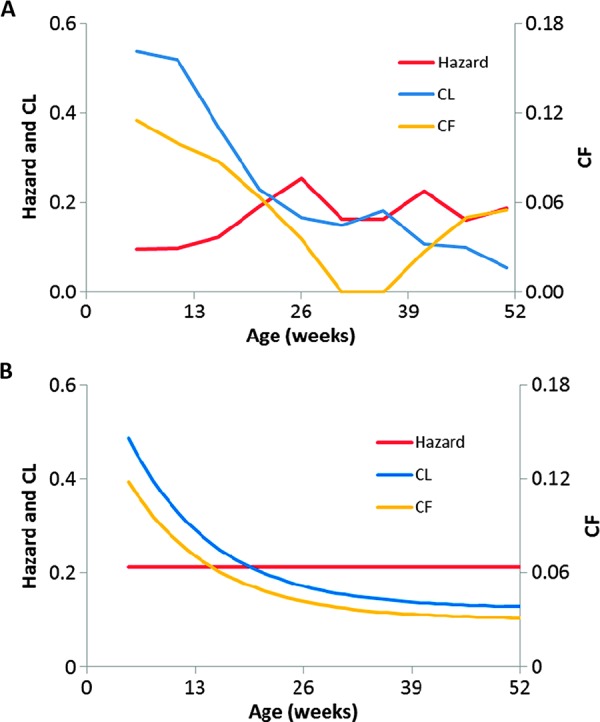
Age-related variation in risks of *T. parva* infection, clinical illness, and death from ECF. (**A**) Empirical estimates of (i) hazard of seroconversion to *T. parva* (*18*), with censoring of non–*T. parva*–related deaths and adjusted for a 14-day delay between infection and a detectable antibody response; (ii) case fatality rate (probability of death conditional on infection, CF); (iii) net clinical rate (probability of death or ECF-like illness conditional on infection, CL). In contrast to hazard, CF and CL both decrease with age (Poisson regression: *F*_1,8_ = 10.4, *P* = 0.012 and *F*_1,8_ = 57.7, *P* < 0.001, respectively). (**B**) Model-predicted estimates for hazard and corresponding predictions for CF and CL with age. Model equations are given in Materials and Methods; parameter estimates are as in Table 3.

Twenty-four ECF deaths (77%) were classified as acute, that is, occurred within 35 days of first infection with *T. parva*. Of the remainder, five were attributed to secondary reinfection with *T. parva*, and two had uncertain infection dates, so these could not be categorized. Of calves surviving infection, 75 (18%) experienced clinical illness consistent with acute ECF but, for the majority, no clinical signs were detected. As previously reported (*14*), surviving first infection with *T. parva* (using seroconversion as a marker of previous exposure) strongly protects against subsequent ECF mortality [hazard ratio, 0.12; 95% confidence interval (CI), 0.07 to 0.22] through the development of T cell–mediated adaptive immunity (*17*). The rate of exposure to *T. parva* estimated from serology data (*18*) was near constant over the first year of life (Fig. 2A). In contrast, both the case fatality rate (proportion dying of acute ECF among those infected) and the net clinical ECF rate (proportion dying and/or experiencing clinical illness) declined with age (Fig. 2A). These patterns are consistent with mathematical model predictions described below (see Fig. 2B).

When calves were infected with the less pathogenic *Theileria* (LPT) species (*T. mutans* and *T. velifera*) before infection with *T. parva*, there were just 2 instances out of 25 (both with *T. mutans* alone) where we observed ECF-like clinical signs (compared with 29 of 53 when infected with *T. parva* alone, a significant difference: Fisher’s exact test, *P* < 0.001). *T. velifera* was never found in the absence of both *T. parva* and *T. mutans*. These data confirm that LPT do indeed have low pathogenicity in this population.

### Natural challenge experiment

To test for evidence of heterologous protection by LPT, we exploited the longitudinal nature of our cohort study to design a large “natural challenge” experiment involving all 310 calves that did not seroconvert to *T. parva* (or die from acute ECF) until >16 weeks old. Of these calves, 169 had seroconverted to *T. mutans* by 16 weeks old, and 141 had not. We asked whether the outcome of subsequent *T. parva* infection, that is, acute ECF death or survival, was associated with serological evidence of exposure to *T. mutans* at 16 weeks. The natural challenge experiment has the advantage that it is considerably larger than would be feasible in a true experiment. However, because *T. mutans* status is not assigned randomly, the approach requires that there is no bias between *T. mutans* seropositive and seronegative calves with respect to factors potentially influencing the ECF case fatality rate. The requirement was met for all key indicators. There was minimal difference in the mean age of subsequent seroconversion to *T. parva* among survivors (32 and 31 weeks for seropositive and seronegative calves, respectively). The two groups had similar levels of infections with strongyle worms at week 16 [median, 325 and 250 eggs per gram (epg), respectively]; this is a possible confounder of the effect of *T. mutans* on clinical outcome of *T. parva* infection (*14*). Finally, there were seven deaths in each group (median age = 41 weeks for both) due to causes other than acute ECF.

**Fig. 3:**
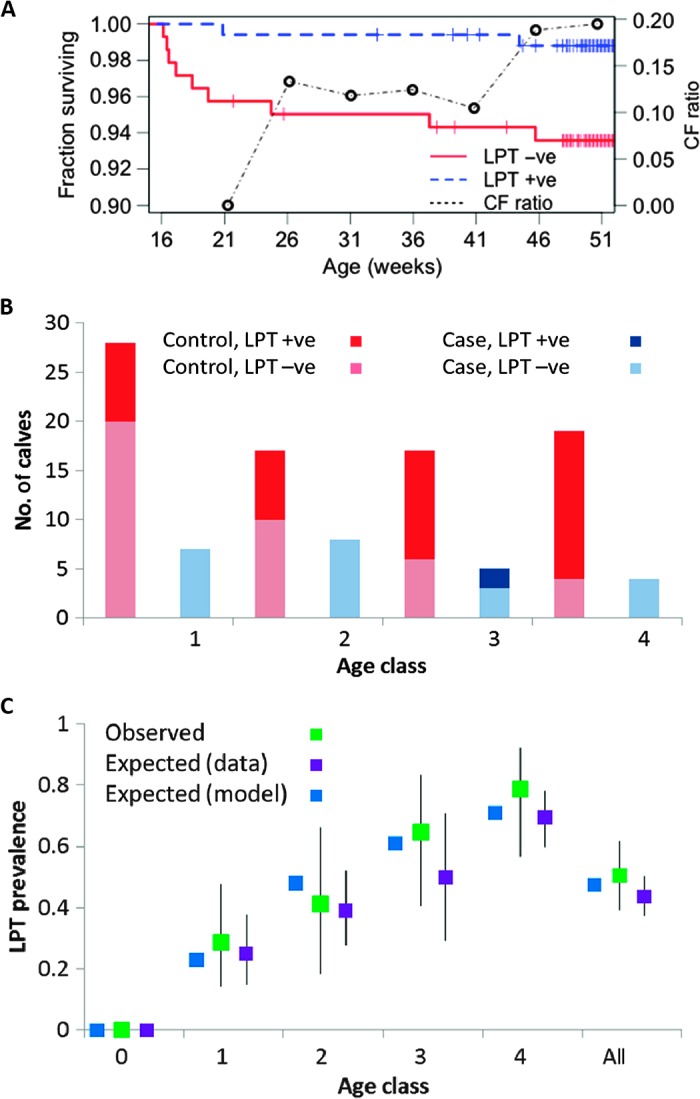
Epidemiology of LPT infections and relationships with clinical outcome of *T. parva* infection. (**A**) Kaplan-Meier plot for calves first infected by *T. parva* at >16 weeks of age. Observed fractions surviving for those initially exposed (*n =* 169) and unexposed (*n =* 141) to LPT are compared. Tick marks indicate censoring (due to non-ECF deaths or end of observation period). A log-rank test indicates a significant difference [χ^2^(1) = 6.2, *P* = 0.013]. The change in overall relative risk for the two groups (CF ratio) as the calves age indicates falling levels of protection and is consistent with model predictions (see table S2). (**B**) Numbers of case and control calves by age class (1 to 4; see Table 1) and detection or nondetection of LPT, having excluded five calves with unknown LPT status. Controlling for age, detection of LPT is significantly protective (odds ratio = 0.11, *P* = 0.002). (**C**) Comparison of observed age-related LPT prevalences at time of first detection of *T. parva* surviving case-control calves (*n =* 81) in different age classes and overall (with 95% CIs) with expected prevalences averaged over all visits when *T. parva* was not detected. Expected prevalences in surviving calves from a mathematical model (see main text) are compared.

Two calves in the *T. mutans* seropositive group died of acute ECF before 1 year of age, compared with nine calves in the *T. mutans* seronegative group (Fig. 3A). The difference in acute ECF mortality rate (Fig. 3A) was statistically significant [log-rank test: χ^2^(1) = 6.2, *P* = 0.013], and the size of the protective effect and the trend to decrease over time (Fig. 3A) were both consistent with the predictions of a mathematical model (see below). This result is consistent with heterologous protection, but serology does not indicate whether the *T. mutans* infection is still active or has been cleared.

### Case-control study

We proceeded to study the infection histories of a subset of the calf cohort using a polymerase chain reaction (PCR)–based test, the reverse line blot (RLB) hybridization assay, which detects active infections (see Materials and Methods) and is better suited than serology to determine infection histories in the youngest calves because of the presence of maternal antibodies. We conducted a nested case-control study using 105 calves: all 24 calves that died of acute ECF (cases) and an age-matched subsample of 81 of the 392 calves that survived exposure to *T. parva* (controls). Cases and controls had mean ages of 113 and 107 days, respectively. These calves were screened for the presence of other species of *Theileria* parasites at every time point between birth and seroconversion to *T. parva* (or death) using RLB. For statistical analysis, we defined four age classes (Fig. 3B and Table 1), noting that the earliest detection of *T. parva* was at 16 days old. Exact conditional logistic regression indicated that concurrent infection with LPT at first infection with *T. parva* was associated with an 89% reduction (95% CI, 47 to 99%; *P* = 0.002) in the odds of dying of ECF (Fig. 3B and Table 2A). The analysis controlled for the increase in LPT prevalence with age, noting that LPT prevalence in calves when they were first infected with *T. parva* was very similar to LPT prevalence in calves of the same age but not infected with *T. parva* (Fig. 3C).

**Table 1:** Case-control study. Age distribution of ECF deaths (cases) and age-matched, *T. parva*–exposed but surviving calves (controls). The conditional logistic regression analysis (see Materials and Methods) controls for any imbalance across age classes. The number of deaths by age class predicted by the mathematical model (see Material and Methods) is compared.

**Age** **class**	**Age** **(days)**	**Observed no. of** **deaths**	**Predicted no. of** **deaths**	**No. of** **survivors**
1	15–59	7	10.5	28
2	60–104	8	5.4	17
3	105–159	5	3.1	17
4	160+	4	5.1	19
All		24		81

**Table 2A:**
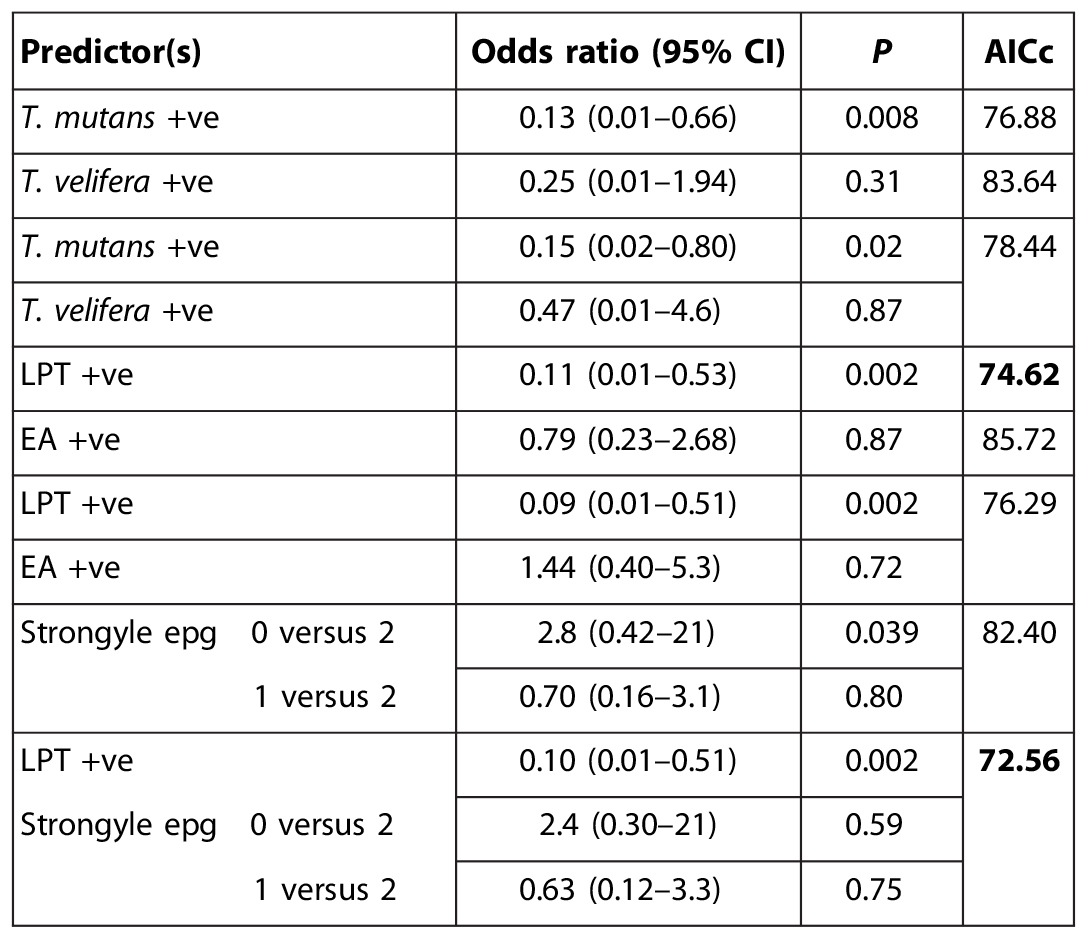
Conditional logistic regression analyses of case-control data. Each row shows results from a model including the variable or variables shown. All models include age stratification (see Table 1). Odds ratios give the effect of the respective predictors on the odds of death by ECF. AICc is the corrected Akaike information criterion. Bold type indicates models with relative probabilities ≥0.05. (A) Co-infection predictors, univariate and bivariate analyses. The bivariate model with both LPT and strongyle epg is the best fit; however, the univariate model with LPT only has a relative probability >0.05 and generates a very similar odds ratio. EA, *Ehrlichia*/*Anaplasma*.

**Table 2B:**
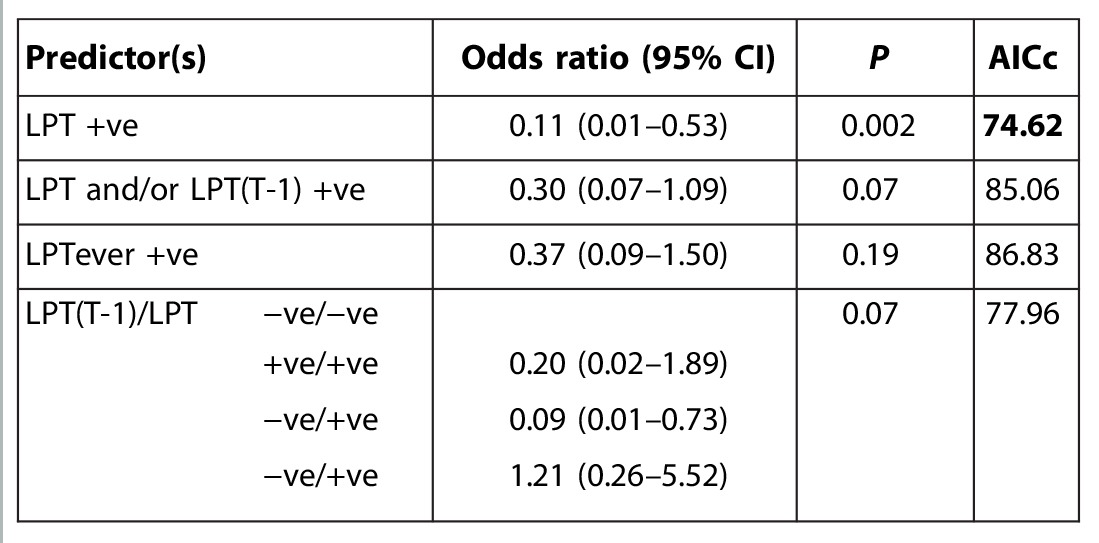
Temporal predictors. LPT indicates status at time T0, LPT(T-1) indicates status at time T-1 (35 days earlier), and LPTever indicates whether infected at any time up to and including T0. In univariate analyses, current LPT status alone is the best predictor; incorporating variables relating to previous LPT status increases the AICc value. Comparing different sequences of LPT status for times T-1 and T0 (−ve/−ve, +ve/+ve, −ve/+ve, +ve/−ve) in a multivariate analysis confirms that protection is associated with LPT-positive status at time T0.

We did not find any association of outcome with other co-infections, for example, strongyle nematodes or the tick-borne rickettsia *Ehrlichia* spp. and *Anaplasma* spp., and the association between outcome and LPT was robust to the inclusion of these as covariates in the analysis (Table 2A). The association was also robust to the inclusion of any of >40 other covariates relating to the calf, its dam, or its environment (see the Supplementary Materials).

Incorporation of previous LPT status in the predictor variable did not improve model fit (Table 2B), indicating that protection was most strongly associated with concurrent LPT infection. Nonetheless, because LPT causes chronic infections, the protective effect may be long-lived. However, if calves do clear LPT infection, then they revert to a level of risk that is statistically indistinguishable from calves that were never infected (odds ratio = 1.21; 95% CI, 0.26 to 5.52; Table 2B).

If ECF mortality were associated with only specific, rare *T. parva* strains, this could explain the low and age-related case fatality rate (although not the association with LPT). However, every individual infection of a subset that was genotyped exhibited a low number of alleles per locus (means of 1.09 to 1.18) together with a low expected heterozygosity (0.029 to 0.178). In contrast, a much higher level of heterozygosity (0.5 to 0.843) was observed in the overall population, calculated on the basis of the predominant multilocus genotypes. We interpret these data as indicating that each calf is infected with a low number of *T. parva* genotypes, and that a single, highly abundant genotype predominates in each calf, but the predominant genotype varies from calf to calf with no obvious clustering of genotypes. So, there is evidence of a high level of diversity present in the *T. parva* population, consistent with previous studies (*19*), but no indication that there is a single pathogenic genotype circulating in the cattle population.

To investigate the physiological effects of concurrent LPT infection, we characterized the initial (pre-seroconversion) response to *T. parva* infection for a subset of 68 calves in the case-control study using a combination of hematological and immunological variables combined in a single index of clinical “severity” (see the Supplementary Materials). Severity, thus defined, was closely associated with whether calves remained healthy or died (table S1). However, calves that became clinically ill had similar severity scores to those that died (fig. S1). Given this observation, we looked for an association between LPT and clinical ECF in all surviving calves. Calves that were clinically ill had an LPT prevalence intermediate between those that remained healthy and those that died (fig. S2). We interpret these results as calves that died being at the extreme end of a clinical spectrum that is strongly influenced by the presence or absence of LPT.

### Mathematical modeling

We used a mathematical model to explore the epidemiological consequences of heterologous protection against infectious disease in an endemic setting. The model (see Materials and Methods) considers the cumulative fraction of hosts of age *a* exposed to a high pathogenicity parasite [*C*(*a*)] and the fraction currently infected with a low pathogenicity parasite [*L*(*a*)] and is potentially applicable to any combination of parasite or pathogen species in any host. Model parameters are defined in Table 3 and were estimated using data from the calf cohort. The key model output is *U*(*a*), the age-specific rate at which hosts are first exposed to high pathogenicity infection in the absence of concurrent low pathogenicity infection, where *U*(*a*) = Λ_H_(1 − *C*(*a*))(1 − *L*(*a*)). These hosts do not benefit from heterologous protection and so have elevated case fatality and net clinical ECF rates. Hosts that were never infected and hosts that have cleared low pathogenicity infections are both included in *U*(*a*). The use of a single force of infection, Λ_L_, for LPT is supported by the highly correlated distributions of LPT species (association test for detection of *T. mutans* and *T. velifera* by RLB at 1 year old: odds ratio = 6.8, χ^2^ = 78.9, *P* < 0.001). The model does not specify the mechanism underlying heterologous protection; this could, in principle, comprise direct competition between parasite species, nonspecific innate immune responses, or cross-reactive adaptive immune responses.

**Table 3:** Parameter definitions and best-fit estimates for mathematical model.

**Parameter**	**Description**	**Source data**	**Central estimate (95% CI)**
Λ_H_	Force of infection with *T. parva*	Fraction ever infected with *T. parva*	0.0352 week^–1^ (0.0315–0.0388)
Λ_L_	Force of infection with LPT	Fraction of *T. parva*–exposed calves ever infected with LPT	0.0685 week^–1^ (0.0576–0.0800)
σ_H_	Rate of loss of *T. parva*	Fraction of calves with *T. parva* infection by RLB at 1 year	0.335 week^–1^ (0.256–0.450)
σ_L_	Rate of loss of LPT	Fraction of calves with LPT infection by RLB at 1 year	0.0234 week^–1^ (0.0194–0.0278)
μ	Fraction of high-risk calves that die	Scaled to observed no. of acute ECF deaths	0.118 (0.079–0.168)
η	Fraction of high-risk surviving calves with clinical ECF	Scaled to observed no. of acute ECF deaths + ECF illness	0.486 (0.415–0.556)

For *Theileria* infections in these calves, best-fit parameter estimates (Table 3) indicate that Λ_L_ is almost 2× greater than Λ_H_ (so that young calves are likely to be infected with LPT before *T. parva*) and that σ_H_ is more than 10× greater than σ_L_ [so that LPT infections have a much longer average duration (1/σ_L_ = 43 weeks) than *T. parva* infections (1/σ_H_ = 3 weeks)]. These results imply that, once infected with LPT, most of the calves will remain infected for most or all their first year, whereas *T. parva* infections are cleared rapidly to below detectable levels, consistent with previous observations (*16*). Our estimates of the scaling parameters μ and η correspond to 12% of high-risk calves (*U*) dying and a further 37% becoming clinically ill, in good agreement with estimated case fatality (CF) and net clinical (CL) rates in the youngest calves (12 and 42%, respectively; Fig. 3A).

The model makes a number of predictions that constitute internal validation tests. First, the model predicts LPT prevalence by age in surviving calves in the case-control study, showing good agreement between prediction and observation (Fig. 3C). Second, the model predicts LPT prevalence at T0 (the period when *T. parva* was first detected), given observed prevalence at T-1 (35 days earlier) as the starting condition (Fig. 3C): the central prediction was 0.45, and the model was an acceptable fit to observed data (prevalence = 0.51; 95% CIs, 0.39 to 0.62; χ^2^ test, *P* = 0.32). Third, the model predicts the age distribution of deaths (Table 1) and provides an acceptable fit to the data [χ^2^(3) = 2.82, *P* = 0.42], although numbers are too low to differentiate reliably from a model without heterologous protection [same comparison: χ^2^(3) = 4.71, *P* = 0.19].

**Fig. 4:**
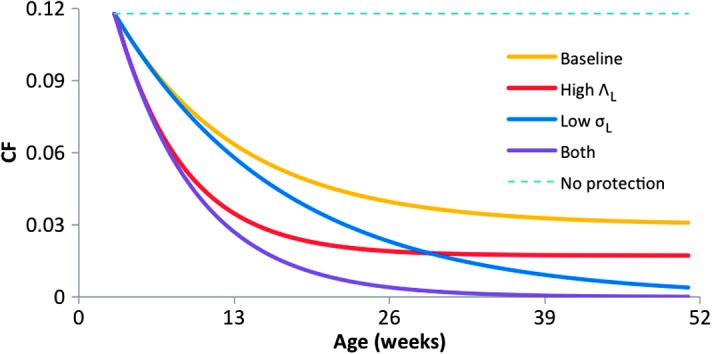
Sensitivity analysis of mathematical model. Sensitivity analysis of model-predicted, age-related changes in the case fatality rate (CF) for different values of the force of infection with LPT (Λ_L_) and the rate of clearance of LPT infections (σ_L_). Λ_L_ and σ_L_ are varied as indicated, baseline and other parameter values as in Table 3, and high Λ_L_ indicates 2× the baseline and low σ_L_ corresponds to a value of zero. CF is constant with age in the absence of heterologous protection by LPT.

Consistent with the field data (Fig. 2A), the model predicts a constant value for the rate of infection with *T. parva*, but marked age-related decreases in both CF and CL (Fig. 2B), more so if the force of infection with LPT (Λ_L_) is high and/or if the rate of clearance of LPT infections (σ_L_) is low (Fig. 4). In the absence of heterologous protection, the model predicts constant CF and CL with age (Fig. 4).

As sensitivity analyses, we compared model-predicted changes in CF with age for different parameter combinations. The results (Fig. 4) illustrate the comparative influence of Λ_L_ and σ_L_ on CF as a function of age: increasing Λ_L_ lowers CF overall, but especially in younger calves; decreasing σ_L_ also lowers CF overall, but especially in older calves. High Λ_L_ and low σ_L_ together result in very low CF in all but the very youngest calves.

Model-predicted, overall fractions of exposed and high-risk calves during the first year of life (*C*_1_ and *U*_1_, respectively) and overall case fatality rate (CF_1_) were compared for three scenarios: (A) varying Λ_L_ alone; (B) varying Λ_H_ alone; (C) varying both Λ_H_ and Λ_L_ by the same factor. All of these could represent natural variation in forces of infection, for example, due to variation in tick densities (*20*), but scenario B also corresponds to *T. parva*–specific interventions (for example, vaccination—see below) and scenario C to nonspecific interventions (for example, tick control using acaricides).

**Fig. 5:**
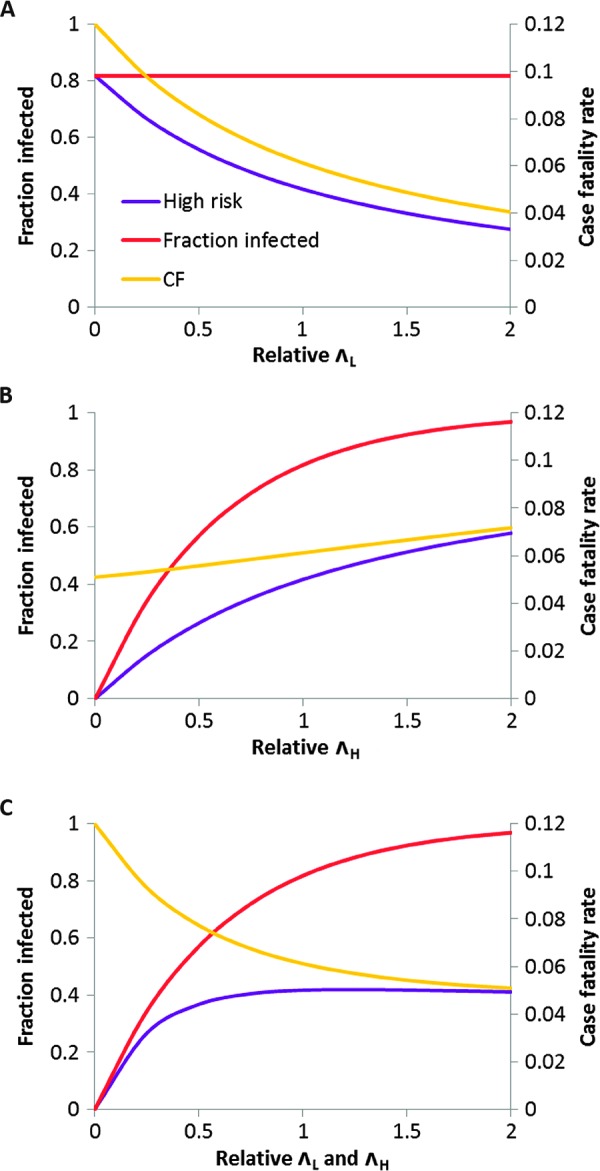
Modeling the effect of LPT infections on clinical burden due to *T. parva*. (**A**) Model outputs for scenario A, changing the force of infection for LPT species, Λ_L_ (relative to baseline, scaled to 1 on horizontal axis), but not changing Λ_H_. Baseline parameter values are as in Table 3. Fraction of calves infected with *T. parva* before 1 year old (unchanged), high-risk infections only, and overall case fatality rate are shown. (**B**) Same outputs for scenario B, changing the force of infection for *T. parva*, Λ_H_, but not changing Λ_L_. (**C**) Same outputs for scenario C, changing both Λ_H_ and Λ_L_. In contrast to scenario B, there is minimal change in the fraction of calves at high risk over a fourfold change (from 0.5 to 2) in relative forces of infection for this set of parameter values.

In scenario A, decreasing Λ_L_ does not change exposure to *T. parva* but substantially increases both CF_1_ (Fig. 5A) and net ECF mortality. When Λ_L_ = 0, 82% of calves are exposed to high-risk *T. parva* infections during their first year of life, and the overall case fatality rate is 12%. In terms of the ECF burden in our study population, if LPT were absent, the model predicts a 75% increase in deaths due to acute ECF (allowing for nonzero mortality in co-infected calves; Fig. 3B), which equates to a 24% increase in total deaths. These results illustrate that we expect variation in ECF burden to reflect the value of Λ_L_ as well as Λ_H_.

In scenario B, a 75% reduction in Λ_H_ only (from 2× to 0.5× baseline) results in a >50% fall in the fraction of calves at high risk, *U*_1_ (Fig. 5B). In contrast, in scenario C, the same reduction in Λ_H_ but coupled to an equivalent reduction in Λ_L_ results in a much smaller (~10%) fall in *U*_1_, but correspondingly higher CF_1_ (given that net exposure to *T. parva* is unchanged; Fig. 5C). For other plausible sets of parameter values, scenario C can result in increases in both *U*_1_ and CF_1_. The model thus provides a novel explanation for the puzzling phenomenon of endemic stability, where substantial variation in the prevalence of *T. parva* infection over a certain level results in little variation in net mortality and morbidity due to ECF (*14*). The model predicts that, as a consequence of heterologous reactivity, endemic stability will result whenever Λ_H_ and Λ_L_ covary (Fig. 5C).

Finally, we considered the effects of changing the prevalence of LPT at different ages, *L*(*a*). A key result (table S2) is that the protective effect observed in the natural challenge experiment (odds ratio = 0.18; 95% CI, 0.02 to 0.87) is consistent with that predicted by the model (odds ratio = 0.29). The model also predicts that the size of the protective effect should diminish over time as some positive calves clear their LPT infections and some negative calves acquire LPT infection (cf. Fig. 3A).

## DISCUSSION

This study provides three strands of evidence for heterologous protection against ECF in a population of indigenous African cattle. The natural challenge analysis indicates that LPT status predicts clinical outcome; the case-control study quantifies the level of protection; and the mathematical model of heterologous protection successfully predicts key features of the epidemiology of ECF. Future work should include experimental studies both to confirm these observations and to determine the mechanisms underlying heterologous protection against ECF by LPT. A working hypothesis is that the well-known immunomodulatory effects of *T. parva* (*17*) are moderated by the presence of LPT, so reducing the severity of infection (noting that we were not able to closely monitor cytokine levels during this study).

Our findings have implications for the epidemiology and control of ECF. The predicted 43% decrease in deaths due to acute ECF due to the presence of LPT represents a substantial contribution of these naturally occurring infections to survival of indigenous cattle, a previously unrecognized “ecosystem service” that compares favorably with the best human efforts to control ECF and contributes to the viability of cattle production in this region. Our analysis also explains why acaricide spraying (which reduces exposure to all *Theileria* species) can fail to reduce disease burden (*21*). The results also suggest that lack of heterologous protection may contribute to the higher ECF case fatality rate among European cattle breeds, which in this region are typically managed so as to reduce their exposure to all tick-borne infections. The only vaccine available against ECF [the ITM (infection and treatment method) vaccine] relies on inoculation with *T. parva* parasites and simultaneous treatment with the antibiotic oxytetracycline (*17*). Our results suggest a novel alternative approach, inoculation of young calves with more benign *T. mutans* or *T. velifera*, without the need for treatment. The chronic nature of these infections and the level of heterologous protection reported here suggest that this would be an effective intervention (table S2), helping calves to survive their first exposure to *T. parva* and develop immunity. Successful ECF control would benefit an estimated 30 million cattle in sub-Saharan Africa, reducing the costs of treatment as well as reducing demand for both antibiotics and acaricides.

Beyond ECF, the generic nature of our model makes its predictions applicable to a range of infectious diseases. *Theileria* is just one of many groups of closely related parasites that can co-infect their hosts and have varying pathogenicities: others include *Plasmodium* spp., *Trypanosoma* spp., *Eimeria* spp., *Schistosoma* spp., *Babesia* spp., and intestinal helminths. For human malaria, in areas where the highly pathogenic *Plasmodium falciparum* co-occurs with less pathogenic species such as *Plasmodium vivax*, our analysis suggests a unified explanation for the apparent failure of bed nets to reduce the burden of childhood disease (*22*), the lack of a correlation between burden and incidence of infection (*23*), and age-related mortality due to severe malaria (*24*), potentially supplanting earlier, disconnected attempts to explain these observations (*21*, *24*). For malaria and other human parasites, a better understanding of the mechanisms underlying heterologous protection may suggest novel ways to prevent clinical disease.

In host-parasite systems, where there is heterologous protection, there are two possible host strategies for avoiding an adverse outcome of infection: (i) being resistant to high-pathogenicity parasites, and/or (ii) being highly susceptible to and not subsequently clearing low-pathogenicity parasites. This implies conflicting selection pressures for and against generic susceptibility to infection, for example, to *Theileria* spp. and their tick vectors in this cattle population. We speculate that the African buffalo (*Syncerus caffer*) has evolved the second strategy against ECF: buffalo are known to support extremely high prevalences and diversity of *T. mutans* and *T. velifera* (*25*), and this may contribute to their very rarely suffering clinical ECF despite high rates of exposure to *T. parva* (see Fig. 4). A similar argument has been made for human malaria in the South Pacific, where a genetic polymorphism (associated with α^+^-thalassemia) that increases susceptibility to *P. vivax* may persist because of the protection this gives against severe disease due to *P. falciparum* (*12*). Both these examples imply counterintuitive adaptive benefits of increased susceptibility to infection by related pathogens.

Our results illustrate a substantial reduction in the risk of morbidity and mortality in parasite infection due to co-infection by congeneric parasites. We suggest that heterologous protection may be an important determinant of the burden and distribution of many parasitic diseases in many host populations, including humans.

## MATERIALS AND METHODS

### Study design

The Infectious Diseases of East African Livestock (IDEAL) project had the overall objective of determining the impact of co-infections on mortality and clinical disease in a birth cohort of indigenous African cattle. The study design is described in detail elsewhere (*26*). Key aspects pertinent to this analysis are set out below.

#### Study population

The study was conducted in a population of East African shorthorn zebu cattle resident in an area in western Kenya falling within a 45-km radius of the study’s field laboratory in the town of Busia at the Kenya-Uganda border. Using a stratified (by agroecological zone) random cluster sampling approach, study animals were selected from smallholder farms in 20 “sublocations” (smallest administrative unit in Kenya) (see Fig. 1). These were mixed crop-livestock smallholder farms averaging 2 ha in size, growing food crops, and keeping an average of five cattle plus other livestock species.

#### Sample size

The inclusion criteria required that the calves were recruited into the study within 7 days of birth and born to a dam that had been on the farm for at least 1 year. Only one calf per farm could be in the study at any one time. Herds practicing stall-feeding and calves from dams artificially inseminated were excluded to avoid recruiting cross-bred animals. We recruited 548 calves that met these criteria. Prophylaxis of any kind was rare in this population, and data from the six calves that received anthelmintic or antibiotic treatment during the study were censored at the time of treatment in accordance with pre-set criteria.

#### Sampling

Recruitment was staggered evenly over a 2-year period (October 2007 to September 2009) and across sublocations to control for any seasonal and spatial variation. Calves were routinely visited at 5-week intervals until leaving the study either at death or at 1 year of age. A complete clinical examination was conducted at the recruitment visit and during each of the 5-week routine visits and whenever clinical illness was reported (see below). Samples including blood smears, whole blood, serum samples, and fecal samples were collected for screening of pathogens (see below) and for measurement of hematological and immunological variables. A complete list of variables recorded is given in the Supplementary Materials. Visits were made until the calf died or left the study at 1 year.

#### Clinical illness

The calves’ owners and/or the resident local animal health assistant were asked to report all episodes of clinical illness (of any nature, including trauma) in the study calves to the project team. These reports were followed up within 24 hours, and the calf was subjected to a detailed examination as above. Every effort was made to standardize reporting of clinical illness (*27*). We note that clinical episodes subsequently diagnosed as ECF (see below) had a very similar age distribution to ECF deaths (Fig. 2A). Reporting and recording of all clinical episodes (and deaths) were blind to the calf’s infection status.

#### Postmortems

For the study animals that died or were euthanized during the study, a complete postmortem examination was carried out by following a standard body system–by–body system veterinary postmortem routine (*15*). The study team reviewed results from parasitological and histological examination of samples collected at postmortem, and gross lesions observed at postmortem, to determine the specific etiological cause of death for each case.

#### Ethics

The study was reviewed and approved by the University of Edinburgh Ethics Committee (reference number OS 03-06) and also by the Institute Animal Care and Use Committee of the International Livestock Research Institute, Nairobi. Standard techniques were used to collect blood and fecal samples for diagnosis and identification of disease and infecting pathogens. The calves were restrained by professional animal health assistants or by qualified veterinary surgeons. A veterinary surgeon was available to examine any calf falling sick during the course of the study. Any calf that was in severe distress due to trauma or disease was humanely euthanized by intravenous injection of sodium pentobarbital by a veterinary surgeon. All participating farmers gave informed consent in their native language before recruiting their animals into the study.

#### *Theileria* diagnostics

Serological tests were performed on samples collected during routine visits to all calves in the study using species-specific enzyme-linked immunosorbent assays (ELISAs) for *T. parva* and *T. mutans* (*18*). As previously (*18*), seroconversion was defined as a rising titer to percent positivity (PP) >20. Seroconversion indicates an infection that occurred at least 14 days previously and may still be active or may have been cleared (*28*).

The RLB hybridization assay was performed on all samples from calves in the case-control study (see below) up to the time of seroconversion to *T. parva*. RLB is a multiplex PCR that tests for a wide range of species of tick-borne parasites of the genera *Theileria*, *Anaplasma*, *Ehrlichia*, and *Babesia* (*29*). This test detects DNA encoding the parasite 18*S* ribosomal RNA gene. The p104 test was carried out on calves where ECF was suspected on clinical grounds. This is a nested PCR test specific to *T. parva* (*30*).

In addition, blood smears were examined for hemoparasites by microscopy. Thin smears were fixed using methanol and stained using Giemsa. Thick smears were directly stained because no fixing was required. One hundred fields were examined under an oil immersion lens. Hemoparasites present were identified to genus level, but it is not possible to distinguish individual species. The level of agreement between the diagnostic tests was explored in detail (see the Supplementary Materials).

#### *T. parva* genotyping

A four-marker profile was generated for 11 samples (nine from calves that died of ECF) using the *T. parva* polymorphic markers MS2, MS8, MS19, and MS21 as described previously (*31*), allowing us to estimate the most abundant multilocus genotype in each calf and the multiplicity of infection.

### Study definitions

For all the analyses reported here, we adopted the following definitions.

*Seroconversion to T. parva* was defined as the ELISA test giving a rising titer to a value >20 PP (*18*). Seroconversion is used solely as a marker of previous exposure to *T. parva*, neither maternal nor serum antibody is protective against infection or disease (*18*).

*First detection of infection* was defined as the earliest visit (routine or in response to clinical episode) at which *T. parva* was detected by one or more of RLB, p104, or ELISA test. We note that infection dates are therefore not known precisely (see below), nor do we have data on infectious dose or on possible superinfection after first infection.

*Acute ECF death* is death attributed to ECF after postmortem examination and occurring less than 35 days after first detection of *T. parva* infection by RLB or p104 test. The 35-day period encompasses most ECF deaths that occur after experimental infection and, by that time, all exposed cattle that survive are expected to have seroconverted (*28*, *32*).

*Clinical ECF* was defined as clinical signs consistent with ECF occurring less than 35 days (*28*) after first detection of *T. parva* infection or within the 35-day period up to seroconversion to *T. parva*. The clinical signs were rectal temperature ≥40°C and enlarged lymph nodes (*16*). In practice, the latter was almost always the parotid lymph node, but for a single calf in the case-control study, ECF was diagnosed on the basis of enlarged precrural and suprascapular lymph nodes.

Although the calves were closely monitored, incomplete ascertainment or reporting of clinical ECF was possible. However, 23 of the 24 acute ECF deaths were preceded by reported clinical illness, suggesting a high level of ascertainment for severe cases. Moreover, there is no reason to suspect ascertainment bias for clinical illness with respect to age or LPT status (the key variables of interest), in which case the only impact of any underreporting would be on the estimate for the parameter η (Table 3).

For *time periods*, T0 was defined as the period encompassing the day of first detection of *T. parva* infection plus any additional visits within the subsequent 34 days. For calves with >1 visit during T0 (*n =* 24, median time interval 11 days), we used variables measured at the first visit that *T. parva* was detected except that LPT was scored as present if detected at any visit up to the point of seroconversion to *T. parva* (to avoid false negatives). This adjustment affected only five calves (four controls, one case) and is conservative with regard to the protective effect of LPT. T-1 was defined as the period encompassing all visits within the 35 days before T0.

We note that our analyses assume that a single measurement of a predictor variable within the first 35 days of a *T. parva* infection is informative. This is appropriate given that we are primarily concerned here with a number of environmental predictors and with chronic co-infections that typically persist much longer than 35 days (that is, LPT or strongyles).

### Statistical analysis

Standard two-by-two association tests were used to look at positive association between variables. Age-related risk of seroconversion was calculated for the entire cohort with calves dying from non–ECF-related causes censored. Changes in case fatality and net clinical rates with age were analyzed using Poisson regression implemented using SAS V9.3.1 PROC GLIMMIX. Survival analysis was carried out using R package 2.37-7.

#### Natural challenge experiment

From the cohort study, we identified all calves that had not seroconverted to *T. parva* by 16 weeks old but seroconverted or died of acute ECF subsequently. We divided these into two groups: those calves that had seroconverted to *T. mutans* by 16 weeks old and those that had not. We compared the history of exposure to *T. parva* and ECF mortality in the *T. mutans* seropositive and *T. mutans* seronegative groups from 16 weeks of age. This analysis uses seropositivity at week 16 as a marker of infection, noting the good agreement between serology and RLB data at that age (see the Supplementary Materials). A possible source of misclassification (false negatives) was exposure to *T. velifera* before 16 weeks of age (because no serological test for *T. velifera* is available). Inspection of the RLB data revealed just a single instance of this, in a surviving calf; adjustment for this marginally strengthens the result.

#### Case-control study

For this analysis, cases were deaths due to acute ECF (as defined above). Controls were selected on the basis of age at seroconversion to give the closest possible match to the age of death of cases. The target control/case ratio was 3:1 (higher ratios give relatively small increases in statistical power). Selection of controls was blind to LPT status. All calves in the case-control study were screened for the presence of different species of *Theileria* parasites at every time point between birth and seroconversion to *T. parva* (or death) using RLB (see above). Ages of first infection with *T. parva* were known only after RLB screening and three putative cases were found not to meet the criteria for “acute” ECF deaths, that is, within 35 days of first infection. Case-control data were analyzed using exact conditional logistic regression controlling for age (using four age classes). This was implemented using SAS V9.3.1 PROC LOGISTIC.

For analysis of co-infections, univariate exact conditional logistic regression was conducted with all co-infections of interest as predictors of outcome in turn, including *T. mutans* positive by RLB, *T. velifera* positive by RLB, EA positive by RLB, and strongyle egg count category (0, 1, or 2; see the Supplementary Materials, noting that counts were sufficiently stable that solitary missing values could reasonably be interpolated if adjacent observations were in the same category). In addition, we used *T. mutans* positive and *T. velifera* positive in a multivariate analysis, and then *T. mutans* and/or *T. velifera* positive as single variable, LPT. Bivariate conditional logistic regression was conducted with LPT and all other co-infection variables individually to check the robustness of the association with LPT. In addition, bivariate conditional logistic regressions were conducted with LPT plus each of the variables relating to the calf’s physical environment, the calf’s dam, and the calf’s status at birth (see above for the full list). To determine the role of previous LPT infection, we defined two additional variables: LPT(T-1), that is, LPT status 35 days earlier (see above), and LPTever, that is, whether infected with LPT at any time up to and including T0. We also considered all four combinations of LPT status at times T0 and T-1 as separate predictors in a multivariate analysis.

For all conditional logistic regression analyses, model fits were compared using the corrected AICc, and probabilities relative to the best model less than 0.05 (equivalent to AICc < minimum AICc + 2.30) were noted.

### Mathematical modeling

#### Model structure

To explore the consequences of heterologous protection at the population level, we used a mathematical model comprising the following coupled differential equations:

$$\frac{dC}{da}=\Lambda_{H}(1-C(a))$$

$$\frac{dL}{da}=\Lambda_{L}(1-L(a))-\sigma_{L}L(a)$$

where *a* is age, *C* is the cumulative fraction of hosts ever exposed to a high-pathogenicity parasite species, and *L* is the fraction currently infected with a low-pathogenicity species. Model parameters are defined in Table 3.

#### Parameter estimation

Using numerical solutions to the model equations, parameters Λ_H_ and Λ_L_ were fitted as point estimates with binomial 95% CIs directly to the fractions of calves that were either seropositive or RLB-positive for *T. parva* and LPT, respectively, at the final visit (at 51 weeks old). Calves were assumed to remain free of infection up to 14 days old, that is, *C* = *L* = 0 for *a* ≤ 2 weeks. Although ELISA tests are not available for *T. velifera*, this species was very rarely encountered in calves that remained free of *T. mutans* (so any correction would be minor and well within estimated CIs). Using the same approach, and given the central estimates of Λ_H_ and Λ_L_, σ_H_ and σ_L_ were fitted directly to the fractions of calves that were RLB-positive for *T. parva* and LPT, respectively, at the final visit. CIs were estimated by fitting to binomial 95% CIs for these fractions. We modeled both LPT species using a single force of infection, Λ_L_; this corresponds to a common transmission route, an assumption supported by the strongly correlated distributions of *T. mutans* and *T. velifera* (see Results) and consistent with their sharing the same tick vector, *Amblyomma* spp. (The effects reported here are even more pronounced if the LPT species are independently transmitted and modeled using separate force of infections.) The parameters μ and η were fitted as point estimates given the observed numbers of acute ECF deaths and of clinical cases/deaths relative to the model-derived estimate of the total number of calves experiencing high-risk infections up to 51 weeks of age, *V*_1_. The model fitting approach assumes that parameters are constant with age, which is consistent with the serological data (see Fig. 2A) for Λ_H_ and Λ_L_, the RLB data for Λ_L_ and σ_L_ (Fig. 3C), and the age distribution of deaths for μ (Table 1), but cannot be tested directly for σ_H_ and η.

#### Model validation

We conducted a series of internal validation tests of the model as follows: (i) predicting LPT prevalence by age in surviving calves in the case-control study; (ii) predicting the age distribution of ECF deaths; and (iii) using observed prevalence at T-1 as the starting conditions, predicting the prevalence at T0 by evaluating the model over a period of 5 weeks.

#### Scenarios and sensitivity analysis

Model-predicted, overall fractions of exposed and high-risk calves during the first year of life (*C*_1_ and *U*_1_, respectively) and overall case fatality rate (CF_1_) were compared for three scenarios: (A) varying Λ_L_ alone, (B) varying Λ_H_ alone, and (C) varying both Λ_H_ and Λ_L_ by the same factor. As a sensitivity analysis, we compared age-related changes in CF as predicted by the mathematical model for four different parameter combinations: (1) baseline parameter values as in Table 3; (2) doubling the force of infection for LPT (Λ_L_); (3) setting the rate of recovery from LPT infection (σ_L_) to zero; and (4) both (2) and (3). We also considered the effects of changing the prevalence of LPT at different ages, *L*(*a*). This was done first to generate an expected effect size for the natural challenge experiment (see above), and second to estimate the expected impact of inoculation with LPT as a control measure. At a given age *a*, *C*(*a*) was set to zero and *L*(*a*) was set to its value using baseline values of the parameters Λ_L_ and σ_L_ or 0 or 1. The expected number of deaths up to 52 weeks was calculated for each scenario, and effect sizes were calculated using odds ratios.

## Supplementary Material

http://advances.sciencemag.org/cgi/content/full/1/2/e1400026/DC1

## SUPPLEMENTARY MATERIALS

Supplementary material for this article is available at http://advances.sciencemag.org/cgi/content/full/1/2/e1400026/DC1 Fig. S1. Relationship of subclinical, clinical, and fatal infections to clinical variables. Fig. S2. Relationship between clinical outcome and LPT prevalence. Table S1. Conditional logistic regression analyses of clinical predictors. Table S2. Impact of setting LPT prevalence at age *a*, *L*(*a*), on subsequent acute ECF death rate. References (*33*, *34*)

## References

[1] Dye C., Williams B. G., The population dynamics and control of tuberculosis. Science 328, 856–861 (2010). 20466923 10.1126/science.1185449

[2] Read A. F., Taylor L. H., The ecology of genetically diverse infections. Science 292, 1099–1102 (2001). 11352063 10.1126/science.1059410

[3] Lello J., Boag B., Fenton A., Stevenson I. R., Hudson P. J., Competition and mutualism among the gut helminths of a mammalian host. Nature 428, 840–844 (2004). 15103373 10.1038/nature02490

[4] Telfer S., Lambin X., Birtles R., Beldomenico P., Burthe S., Paterson S., Begon M., Species interactions in a parasite community drive infection risk in a wildlife population. Science 330, 243–246 (2010). 20929776 10.1126/science.1190333 PMC3033556

[5] Hotez P. J., Kamath A., Neglected tropical diseases in sub-Saharan Africa: Review of their prevalence, distribution, and disease burden. PLOS Negl. Trop. Dis. 3, e412 (2009). 19707588 10.1371/journal.pntd.0000412 PMC2727001

[6] Shrestha S., Foxman B., Weinberger D. M., Steiner C., Viboud C., Rohani P., Identifying the interaction between influenza and pneumococcal pneumonia using incidence data. *Sci. Transl. Med*. 5, 191ra84 (2013). 10.1126/scitranslmed.3005982 PMC4178309 23803706

[7] Graham A. L., Ecological rules governing helminth-microparasite coinfection. Proc. Natl. Acad. Sci. U.S.A. 105, 566–570 (2008). 18182496 10.1073/pnas.0707221105 PMC2206576

[8] Sharma S., Thomas P. G., The two faces of heterologous immunity: Protection or immunopathology. J. Leukoc. Biol. 95, 405–416 (2014). 24212098 10.1189/jlb.0713386 PMC3923083

[9] Welch R. M., Che J. W., Brehm M. A., Selin L. K., Heterologous immunity between viruses. Immunol. Rev. 235, 244–266 (2010). 20536568 10.1111/j.0105-2896.2010.00897.x PMC2917921

[10] Fine P. E. M., Variation in protection by BCG: Implications of and for heterologous immunity. Lancet 346, 1339–1345 (1995). 7475776 10.1016/s0140-6736(95)92348-9

[11] G. S. Nelson, in *Parasitic Zoonoses Clinical and Experimental Studies*, E. J. L. Soulsby, Ed. (Academic Press, New York, 1974), pp. 273–285.

[12] Williams T. N., Maitland K., Bennett S., Ganczakowski M., Peto T. E. A., Newbold C. I., Bowden D. K., Weatherall D. J., Clegg J .B., High incidence of malaria in α-thalassaemic children. Nature 383, 522–525 (1996). 8849722 10.1038/383522a0

[13] Graham A. L., Lamb T. J., Read A. F., Allen J. E., Malaria-filaria coinfection in mice makes malarial disease more severe unless filarial infection achieves patency. J. Infect. Dis. 191, 410–421 (2005). 15633101 10.1086/426871

[14] Thumbi S. M., de C. Bronsvoort B. M., Poole E. J., Kiara H., Toye P. G., Mbole-Kariuki M. N., Conradie I., Jennings A., Handel I. G., Coetzer J. A. W., Steyl J. C. A., Hanotte O., Woolhouse M. E. J., Parasite co-infections and their impact on survival of indigenous cattle. PLOS One 9, e76324 (2014). 24586220 10.1371/journal.pone.0076324 PMC3930515

[15] Thumbi S. M., de C. Bronsvoort B. M., Kiara H., Toye P. G., Poole J., Ndila M., Conradie I., Jennings A., Handel I. G., Coetzer J. A. W., Steyl J., Hanotte O., Woolhouse M. E. J., Mortality in East African shorthorn zebu cattle under one year: Predictors of infectious-disease mortality. BMC Vet. Res. 9, 175 (2013). 24010500 10.1186/1746-6148-9-175 PMC3848692

[16] J. A. W. Coetzer, R. C. Tustin, Eds. *Infectious Diseases of Livestock* (Oxford Univ. Press, Cape Town, ed. 2, 2004), vol. 1.

[17] Morrison W. I., McKeever D., Current status of vaccine development against *Theileria* parasites. Parasitology 133, S169–S187 (2006). 17274845 10.1017/S0031182006001867

[18] Kiara H., Jennings A., de C. Bronsvoort B. M., Handel I. G., Mwangi S. T., Mbole-Kariuki M., Conradie van Wyk I., Poole E. J., Hanotte O., Coetzer J. A. W., Woolhouse M. E. J., Toye P. G., A longitudinal assessment of the serological response to *Theileria parva* and other tick-borne parasites from birth to one year in a cohort of indigenous calves in western Kenya. Parasitology 141, 1289–1298 (2014). 24838078 10.1017/S003118201400050X PMC4113304

[19] Oura C. A. L., Asiimwe B. B., Weir W., Lubega G. W., Tait A., Population genetic analysis and sub-structuring of *Theileria parva* in Uganda. Mol. Biochem. Parasitol. 140, 229–239 (2005). 15760662 10.1016/j.molbiopara.2004.12.015

[20] Cumming G. S., Using habitat models to map diversity: pan-African species richness of ticks (Acari: Ixodida). J. Biogeogr. 27, 427–440 (2000).

[21] Coleman P. G., Perry B. D., Woolhouse M. E. J., Endemic stability—A veterinary idea applied to human public health. Lancet 357, 1284–1286 (2001). 11418173 10.1016/s0140-6736(00)04410-x

[22] Bruce M. C., Macheso A., Kelly-Hope L. A., Nkhoma S., McConnachie A., Molyneaux M. E., Effect of transmission setting and mixed species infections on clinical measures of malaria in Malawi. PLOS One 3, e2775 (2008). 18648666 10.1371/journal.pone.0002775 PMC2467490

[23] Okiro E. A., Al-Taiar A., Reyburn H., Idro R., Berkley J. A., Snow R. W., Age patterns of severe paediatric malaria and their relationship to *Plasmodium falciparum* transmission intensity. Malar. J. 8, 4 (2009). 19128453 10.1186/1475-2875-8-4 PMC2630996

[24] Ross A., Maire N., Molineaux L., Smith T., An epidemiologic model of severe morbidity and mortality caused by *Plasmodium falciparum*. Am. J. Trop. Med. Hyg. 75, S63–S73 (2006). 10.4269/ajtmh.2006.75.63 16931817

[25] Mans B. J., Pienaar R., Latif A. A., Potgieter F. T., Diversity in the 18S SSU rRNA V4 hyper-variable region of *Theileria* spp. in Cape buffalo (*Syncerus caffer*) and cattle from southern Africa. Parasitology 138, 766–779 (2011). 21349232 10.1017/S0031182011000187

[26] de C. Bronsvoort B. M., Thumbi S. M., Poole E. J., Kiara H., Tosas Auguet O., Handel I. G., Jennings A., Conradie I., Mbole-Kariuki M. N., Toye P. G., Hanotte O., Coetzer J. A. W., Woolhouse M. E. J., Design and descriptive epidemiology of the Infectious Diseases of East African Livestock (IDEAL) project, a longitudinal calf cohort study in western Kenya. BMC Vet. Res. 9, 171 (2013). 24000820 10.1186/1746-6148-9-171 PMC3847666

[27] Conradie van Wyk I., Goddard A., de C. Bronsvoort B. M., Coetzer J. A. W., Handel I. G., Hanotte O., Jennings A., Lesosky M., Kiara H., Thumbi S. M., Toye P., Woolhouse M. W., Penzhorn B. L., The impact of co-infections on the haematological profile of East African Short-horn Zebu calves. Parasitology 141, 374–388 (2014). 24553080 10.1017/S0031182013001625 PMC4021814

[28] Radley D. E., Brown C. G. D., Burridge M. J., Cunningham M. P., Peirce M. A., Purnell R. E., East Coast fever: Quantitative studies of *Theileria parva* in cattle. Exp. Parasitol. 36, 278–287 (1974). 4213046 10.1016/0014-4894(74)90067-8

[29] Gubbels J. M., de Vos A. P., van der weide M., Viseras J., Schouls L. M., de Vries E., Jongejan F., Simultaneous detection of bovine *Theileria* and *Babesia* species by reverse line blot hybridization. J. Clin. Microbiol. 37, 1782–1789 (1999). 10325324 10.1128/jcm.37.6.1782-1789.1999 PMC84950

[30] Odongo D. O., Sunter J. D., Kiara H. K., Skilton R. A., Bishop R. P., A nested PCR assay exhibits enhanced sensitivity for detection of *Theileria parva* infections in bovine blood samples from carrier animals. Parasitol. Res. 106, 357–365 (2009). 19902251 10.1007/s00436-009-1670-z

[31] Oura C. A. L., Odongo D. O., Lubega G. W., Spooner P. R., Tait A., Bishop R. P., A panel of microsatellite and minisatellite markers for the characterisation of field isolates of *Theileria parva*. Int. J. Parasitol. 33, 1641–1653 (2003). 14636680 10.1016/s0020-7519(03)00280-7

[32] Ndungu S. G., Brown C. G. D., Dolan T. T., In vivo comparison of susceptibility between *Bos indicus* and *Bos taurus* cattle types to *Theileria parva* infection. *Onderstepoort. J. Vet. Res*. 72, 13–22 (2005). 15991702

[33] Mbole-Kariuki M. N., Sonstegard T., Orth A., Thumbi S. M., de C. Bronsvoort B. M., Kiara H., Toye P., Conradie I., Jennings A., Coetzer K., Woolhouse M. E. J., Hanotte O., Tapio M., Genome-wide analysis reveals the ancient and recent admixture history East African Shorthorn Zebu (EASZ) from Western Kenya. Heredity 113, 297–305 (2014). 24736786 10.1038/hdy.2014.31 PMC4181064

[34] Murray G. G. R., Woolhouse M. E. J., Tapio M., Mbole-Kariuki M. N., Sonstegard T. S., Thumbi S. M., Jennings A. E., Conradie van Wyk I., Chase-Topping M., Kiara H., Toye P., Coetzer K., de C. Bronsvoort B. M., Hanotte O., Genetic susceptibility to infectious disease in East African Shorthorn Zebu: A genome-wide analysis of the effect of heterozygosity and exotic introgression. BMC Evol. Biol. 13, 246 (2013). 24209611 10.1186/1471-2148-13-246 PMC3828575

