# Supplementary material for: Co-infections determine patterns of mortality in a population exposed to parasite infection
Source: Sci Adv. 2015 Mar 20;1(2):e1400026. doi: 10.1126/sciadv.1400026 (PMC4643819; doi:10.1126/sciadv.1400026)
Supplement: http://advances.sciencemag.org/cgi/content/full/1/2/e1400026/DC1 [file supp_1_2_e1400026__index.html]

Science Advances | Science Advances

## Supplementary Materials

**This PDF file includes:**

- Fig. S1. Relationship of subclinical, clinical, and fatal infections to clinical variables.
- Fig. S2. Relationship between clinical outcome and LPT prevalence.
- Table S1. Conditional logistic regression analyses of clinical predictors.
- Table S2. Impact of setting LPT prevalence at age *a, L(a)*, on subsequent acute ECF death rate.
- References (*33, 34*)

Download PDF

**Files in this Data Supplement:**

- Adobe PDF - e1400026\_SM.pdf
