# Supplementary material for: Co-infections determine patterns of mortality in a population exposed to parasite infection
Source: Sci Adv. 2015 Mar 20;1(2):e1400026. doi: 10.1126/sciadv.1400026 (PMC4643819; doi:10.1126/sciadv.1400026)
Supplement: http://advances.sciencemag.org/cgi/content/full/1/2/e1400026/DC1 [file e1400026_SM.pdf]

Science Advances

www.advances.sciencemag.org/cgi/content/full/1/2/e1400026/DC1

Supplementary Materials for

Co-infections determine patterns of mortality in a population exposed to parasite infection

Mark E. J. Woolhouse,* Samuel M. Thumbi, Amy Jennings, Margo Chase-Topping, Rebecca Callaby, Henry Kiara, Marinda C. Oosthuizen, Mary N. Mbole-Kariuki, Ilana Conradie, Ian G. Handel, E. Jane Poole, Evalyne Njiiri, Nicola E. Collins, Gemma Murray, Miika Tapio, Olga Tosas Auguet, Willie Weir, W. Ivan Morrison, Loeske E. B. Kruuk, B. Mark de C. Bronsvoort, Olivier Hanotte, Koos Coetzer, Philip G. Toye

Published 20 March 2015, *Sci. Adv.* **1**, e1400026 (2015)
DOI: 10.1126/sciadv.1400026

This PDF file includes:

Fig. S1. Relationship of subclinical, clinical, and fatal infections to clinical variables.
Fig. S2. Relationship between clinical outcome and LPT prevalence.
Table S1. Conditional logistic regression analyses of clinical predictors.
Table S2. Impact of setting LPT prevalence at age a, $L(a)$, on subsequent acute ECF death rate.
References (*33*, *34*)

Supplementary Materials

Data collected

For each calf in the cohort information was collected on the calf's environment, genotype and infection and health status (*27*). Single observations were made of:

- farmer's age (years)
- farmer's gender (f/m)
- farmer attended school (yes/no)
- farmer's main occupation (farming/other)
- herd size (no. cattle)
- land area (ha)
- elevation (m)
- cattle kept in stall or kraal (yes/no)
- cattle tethered (yes/no)
- cattle watered on farm (yes/no)
- cattle given supplementary feed (yes/no)
- dam antibody titres (percent positivity) against:
    - *Theileria parva*
    - *T. mutans*
    - *Anaplasma marginale*
    - *Babesia bigemina*
- dam body condition score (scale 1-10)
- calf sex (f/m)
- birth weight (kg)

calf genotype, as determined by SNP data from the Illumina© BovineSNP50 v.1 beadchip and used to calculate genome-wide homozygosity and percent introgression with European taurine genome determined using STRUCTURE analysis (*33*,*34*).

Observations made at 5-weekly intervals were:

normalised difference vegetation index (NDVI) at sublocation level

recorded application (yes/no) to any other herd members of:

tick control

worm control

trypanosome control

any vaccine

calf still suckling (yes/no)

calf heart girth measurement (cm)

Pathogen screening at 5-weekly intervals and clinical episodes comprised:

physical examination for ticks (presence/absence):

R. appendiculatus

Amblyomma spp.

microscopy for:

Trypanosoma spp. (presence/absence)

Theileria spp. (levels 0 to 3 where level 1 = one parasite or infected cell per two to ten fields; level 2 = one to 10 parasites or infected cells in every field; level 3 = >10 parasites or infected cell in every field)

flotation method detection (presence/absence in faeces) of:

Coccidia spp.

Strongyloides eggs

strongyle eggs

Trichuris spp.

Toxocara vitulorum

sedimentation technique detection (presence/absence in faeces) of:

Calicophoron spp.

Fasciola spp.

direct Baermann's technique detection (presence/absence in faeces) of *Dictyocaulus viviparous*

faecal larval cultures (presence/absence) for:

Haemonchus placei

Oesophagostomum radiatum

Trichostrongylus axei

Cooperia spp.

faecal egg counts by McMaster method for strongyles (as 3 categories of egg count: 0, 1-499, 500+ per g faeces)

antibody titres (percent positivity) against:

T. parva

T. mutans

A. marginale

B. bigemina

Clinical observations carried out at 5-weekly visits and clinical episodes comprised:

calf showing signs of clinical illness (yes/no)

calf rectal temperature (< 40°C or ≥ 40°C)

lymph node size (normal vs enlarged)

haematology read-outs based on automated blood cell analysis using the pocH-100iV Diff (Sysmex© Europe GMBH) system of:

total serum protein, TSP (g/dl, measured from 100 μL serum using a refractometer (model RHC-200ATC, Westover Scientific))

packed cell volume, PCV (percent, measured manually using a Hawksley microhaematocrit reader)

white blood cells, WBC (count per μl)

platelets, PLT (count per μl)

cytokine levels (during acute *T. parva* infection in the case-control study calves only):

Bovine Transforming Growth Factor β1 ELISA Kit, Cat. No. CSB-E14208B for TGFβ1 (as optical density)

Bovine Interleukin 10 ELISA Kit, Cat No. CSB-E12917B for IL10 (sign-reversed optical density as this is an inhibition assay whereas the TGFβ1 test is a capture assay).

Comparison of diagnostic test results

The performance of the serological tests, PCR-based tests and microscopy for detecting *Theileria* species were compared as follows:

RLB vs serology. Of the 105 calves in the case-control study, 100 were *T. parva* positive by RLB and/or serology (for the remainder ECF was only diagnosed at post mortem). For 82% of these calves, infection was first detected by RLB. Those first detected by serology alone were all subclinical infections. Seroconversion appeared delayed for calves that were RLB positive at 6 weeks.

Of the case-control study calves 88 were *T. mutans* positive by RLB and/or serology. *T. mutans* infection was first detected by RLB in 93% of these. We also explicitly compared *T. mutans* status at T0 (see Materials and Methods) by RLB and serology in order to evaluate serology as a marker of current infection. For 100 of the calves in the case-control study, there was 79% agreement; however, for all but one of the nine RLB negative/serology positive calves the data were consistent with there being an older infection that had been cleared, and for all but one of the 12 RLB positive/serology negative calves the

data were consistent with the infection being too recent for seroconversion to have occurred. This suggests a level of agreement between serology and current *T. mutans* infection in the range 79-98%.

RLB vs p104. No calf was positive by p104 that was never positive by RLB. However, for two calves the age of first infection with *T. parva* was moved forward by the p104 test (by 15 and 16 days).

RLB vs microscopy. There were 376 samples that were *Theileria* positive by RLB and/or microscopy. Of these, 311 (83%) were positive by RLB. There were 29 calves that were RLB negative but positive by microscopy on at least one occasion prior to seroconversion to *T. parva*. These calves were not at altered risk of fatal or clinical ECF (association tests, $P>0.25$).

Sensitivity and repeatability. We re-tested 62 samples that were RLB positive for *T. parva* but negative for other *Theileria* spp. using 5x standard concentrations of primers. All samples were positive for *T. parva* on re-test and only one sample was positive other *Theileria* spp. (for *T. mutans* only), a high level of repeatability that does not suggest any issues with test sensitivity for other *Theileria* species in the presence of *T. parva*. We note that experimental studies have established the capacity of RLB to detect all *Theileria* species in mixed infections (*19*) and show no evidence for displacement of an existing infection by a subsequent infection (*19,31*).

The above results suggest that the sensitivity of the RLB test is in the range 80-90%. The protective effect reported in the main text is robust to imperfect sensitivity in the observed range: if RLB test sensitivity is assumed to be 90% and the number of LPT positives scaled accordingly we obtain odds ratio=0.18, $P=0.004$; assuming 80% sensitivity we obtain odds ratio=0.25, $P=0.011$.

Clinical severity

Analysis of the relationship between outcome and haematological and immunological variables were confined to case-control study calves where *T. parva* was detected prior to seroconversion, i.e. 19 cases and 49 controls. Included variables were: white blood cell count (WBC), total serum protein (TSP), packed cell volume (PCV), platelet count (PLT) ($\log_{10}$ transformed), IL10 and TGFβ1 (see above), all

measured at T0. Univariate conditional logistic regression analyses were used to explore the relationships between each of these variables and acute ECF death (Table S1). In addition, principal components analysis (PCA) was applied to haematological and immunological data. PCA was implemented using PC-ORD software version 6.03 (MJM software Design, Gleneden Beach, OR). The first three principal components were significant ($p<0.05$) and explained 41%, 31% and 17% of the total variance respectively (Fig. S1). Four variables were loading (>0.4) on PCA1 (PCV, logPLT, TSP, IL10), with WBC and TGFβ1 loading (>0.4) on PCA2 and PCA3 respectively. As the principal components are not equally important in measuring overall clinical severity, PCA1-3 were combined to create a single composite index. Using the proportion of these percentages as weights on the score coefficients, a non-standardized index (NSI) was calculated using the following formula:

NSI = (40.6/88.3)x(Factor 1 score) + (30.8/88.3)x(Factor 2 score) + (16.9/88.3)x(Factor 3 score).

The non-standardized 3-axis index (NSI3) from the PCA was the single best predictor of acute ECF death (Table S1) and was used as an index of clinical "severity" in subsequent analyses. The fit of a univariate model of parasite load is also reported in Table S1. This indicates that high visible parasite load in the blood is associated with increased mortality risk, as previously reported (*16,17*).

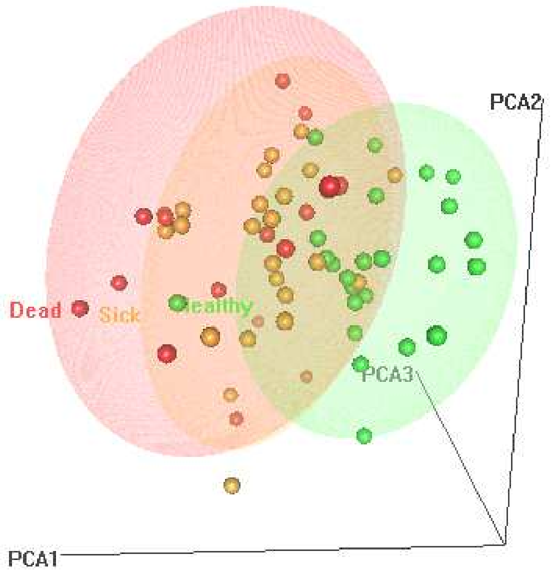

See file **FigureS1animation.avi** for rotating view

Fig. S1. Relationship of subclinial, clinical, and fatal infections to clinical variables. 3D scatterplot and 80% confidence ellipses (R pgl package scatterplot 3D) illustrating the results of the principal components analysis on a subset of *N*=68 calves using six variables: platelet count (PLT, log transformed); total serum protein (TSP); packed cell volume (PCV), white blood cell count (WBC); and levels of the cytokines IL10 and TGFβ1. There were three significant principal components: PCA1, PCA2 and PCA3 which explained 40.6%, 30.8% and 16.9% of the total variation in the data respectively. Individual data points and confidence ellipses distinguish calves that died (red), had clinical ECF but survived (orange), and remained healthy throughout (green).

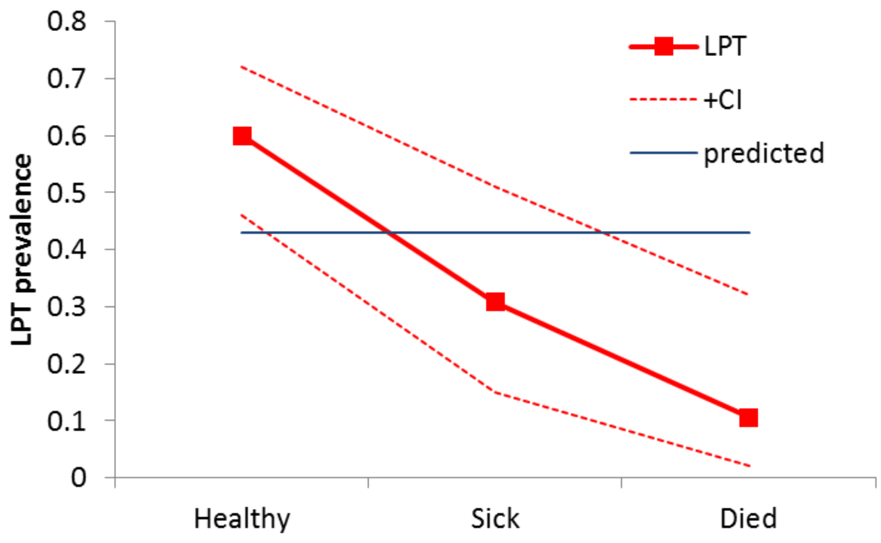

Fig. S2. Relationship between clinical outcome and LPT prevalence. All *T. parva* seronegative calves from the case-control study are grouped into those that died (*N*=19), experienced clinical illness (*N*=26) and those that remained healthy (*N*=37). Observed LPT prevalence in surviving calves (with 95% CIs) is compared with model-predicted LPT prevalence (see Fig. 3C). Overall, surviving calves have close to expected LPT prevalence, but the presence or absence of LPT partly sorts the calves into healthy or sick respectively (general linear model, overall model: $p<0.001$, post hoc test for differences between clinically ill and dead: $p=0.32$).

Table S1. Conditional logistic regression analyses of clinical predictors. Each row shows results from a univariate model with the variable shown for *N*=68 calves. All models include age stratification (see Table 1). Odds ratios give the effect of the predictor on the odds of death by ECF. AICc is the corrected Akaike Information Criterion. Bold type indicates models with relative probabilities ≥ 0.05. Statistics derived from principal components analysis used as predictors are: the first principal component (PCA1); and the non-standardized 2- and 3-axis indices (NSI2 and NSI3 respectively). NSI3 is the best predictor but packed cell volume, total serum protein and PCA1 all have relative probabilities >0.05. A univariate model with parasite load is compared.

**Predictor**	**Odds ratio (95% CIs)**	***P* value**	**AICc**
Packed cell volume	0.81 (0.71-0.93)	0.002	**52.20**
White blood cell count	0.82 (0.68-0.99)	0.043	62.00
Platelet count	0.11 (0.02-0.76)	0.025	59.02
Total serum protein	0.17 (0.06-0.56)	0.003	**50.67**
IL10	0.61 (0.10-3.78)	0.59	66.65
TGFβ1	1.04 (0.84-1.28)	0.76	66.83
PCA1	2.19 (1.33-3.60)	0.002	**51.37**
NSI2	4.33 (1.64-11.5)	0.003	52.97
NSI3	4.85 (1.85-12.8)	0.001	**50.44**
Parasite load (0/1 vs 2/3)	0.13 (0.03-0.53)	0.004	54.46

Table S2. Impact of setting LPT prevalence at age *a*, *L*(*a*), on subsequent acute ECF death rate. Predicted acute ECF death rates are compared for different ages and three different scenarios: all infected, *L*(*a*)=1; none infected, *L*(*a*)=0, and *L*(*a*) as expected using baseline model parameters (Table 3). Odds ratios (comparing *L*(*a*)=1 against the reference value) are shown in italics. For comparison, the equivalent odds ratio from the natural challenge experiment (1 vs 0, 16 weeks) is 0.18 (95% CIs = 0.02-0.87).

**Baseline age, *a***	*6 weeks*	*11 weeks*	*16 weeks*
***L*(*a*) = 0**	0.049	0.048	0.047
***L*(*a*) = baseline**	0.045	0.036	0.030
***L*(*a*) = 1**	0.016	0.015	0.014
odds ratio: 1 vs 0	0.32	0.30	0.29
odds ratio: 1 vs baseline	0.35	0.41	0.46

Data files and biological samples

The data used in the analyses reported here are openly accessible at http://datashare.is.ed.ac.uk/. The data are presented in a single file, IDEALSciAdvdata.csv. The data comprise records of observations made at every visit to all 548 calves included in the IDEAL study cohort and identifies the calves and visits that contribute to each of the major analysis described in the main text.

In addition, we have placed biological samples (of sera and, where appropriate, other tissues) collected from the calves in a repository. The samples can be linked to the data files (via the calf and visit ID numbers) and are available to researchers on request (subject to international shipping regulations). Details can be found at http://azizi.ilri.cgiar.org/.
